# Molecular Mechanism of the Flavonoid Natural Product Dryocrassin ABBA against *Staphylococcus aureus* Sortase A

**DOI:** 10.3390/molecules21111428

**Published:** 2016-10-26

**Authors:** Bing Zhang, Xiyan Wang, Lin Wang, Shuiye Chen, Dongxue Shi, Hongsu Wang

**Affiliations:** 1 College of Food Science and Engineering, Jilin University, Changchun 130062, China; emerald_bing@sina.com; 2 College of Veterinary Medicine, Jilin University, Changchun 130062, China; wxyvip16@163.com (X.W.); wanglin1020@jlu.edu.cn (L.W.); 18443158548@163.com (S.C.); m15754305546@163.com (D.S.)

**Keywords:** *Staphylococcus aureus*, sortase A, dryocrassin, molecular simulations

## Abstract

The intractability of bacterial resistance presents a dilemma for therapies against *Staphylococcus aureus* (*S. aureus*) infection. Effective anti-virulence strategies are urgently needed, reflecting the proliferation of resistant strains. Inhibitors of sortase A (SrtA), enzymes that anchor virulence-related surface proteins, are regarded as promising candidates for countermeasures against bacterial infections. In the present study, the inhibitory effect of dryocrassin ABBA (ABBA) against SrtA and its molecular basis has been examined. Fluorescence resonance energy transfer (FRET) assays were used to determine the inhibitory activity of ABBA against SrtA. To identify the mechanism underlying this activity, molecular dynamics simulations and mutagenesis assays were applied, and the results revealed that the direct engagement of SrtA via ABBA through binding to V166 and V168 significantly attenuated the catalytic activity of SrtA. Taken together, these findings indicated that ABBA is a potential novel antimicrobial agent for *S. aureus* infection via targeting SrtA.

## 1. Introduction

*Staphylococcus aureus* (*S. aureus*), a Gram-positive bacteria identified in the 1880s, is the main etiological agent of multiple infectious diseases, playing a crucial role in skin and surgical site infections [1], simultaneously causing infective endocarditis, pleuropneumonia, osteomyelitis and bacteremia [2]. Several factors, including extensive spread, substance abuse, the improper use of antimicrobials, the tendency of globalization in the fields of politics and economics giving rise to the increasing variation of bacteria, as well as the rapidly growing spectrum of antimicrobial-resistant bacteria, have been suggested to explain the increase in *S. aureus* infection [3,4,5]. The treatment options for the *S. aureus* infections are much more difficult than ever, and new effective strategies against this bacterial infection are needed. 

Recently, an alternative anti-virulence strategy has emerged [6,7,8,9]. Cell surface proteins are vital virulence factors in *S. aureus* infections that enable bacteria to adhere, colonize and interact with their surrounding environment. A majority of surface proteins that covalently bond to cell wall peptidoglycans are anchored through the same “housekeeping” enzyme, sortase A (SrtA) [10]. The proteins anchored through SrtA have the same C-terminal sorting signal, comprising an LPXTG motif (leucine, proline, any amino acid, threonine, and glycine), a hydrophobic domain and a tail of positively charged amino acids [11,12,13]. The peptide bond between the T (threonine) and G (glycine) residues of the motif is cleaved via SrtA [14]. Subsequently, the liberated carboxyl group of threonine and the amino group of the pentaglycine cell wall cross-bridges are linked together through amide bonds, thereby tethering the C-terminal end of the polypeptide chain to the bacterial cell wall envelope [15,16,17]. In previous studies, Cys184, His120, Arg197, and Thr183 have been determined as indispensable in this process. Indeed, when alanine is substituted for any of the residues mentioned above, enzymatic activity decreases more than 1000-fold [18]. Many studies have shown that the virulence of some SrtA mutants significantly decreased in mouse models, and a partial loss of pathogenicity, such as lethal sepsis or abscesses, was also observed in some cases of staphylococcal infections [19,20,21]. Notably, bacterial virulence factors could be ideal targets that hinder *S. aureus* infection [22], making SrtA a potential target.

**Figure 1:**
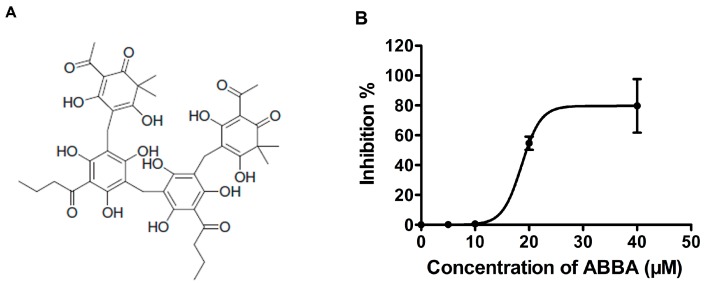
ABBA inhibits SrtA activity. (**A**) The chemical structure of ABBA; (**B**) The effect of ABBA on *S. aureus* SrtA activity. Following pre-incubation with various concentrations of ABBA, the model substrate peptide Dabcyl-QALPETGEE-Edans was added to measure the catalytic activity of each sample using a microplate reader.

In the present study, the natural compound dryocrassin ABBA (ABBA) (Figure 1A) which has been already described as antiviral against flu virus [23], was identified as an effective SrtA inhibitor based on the results of fluorescence resonance energy transfer (FRET) assays. To further determine the molecular mechanism of ABBA against SrtA, molecular dynamics simulations and mutation assays were performed to examine the SrtA-ABBA complex. ABBA, as a potential inhibitor of SrtA enzyme activity, could also be considered a candidate antimicrobial agent for *S. aureus* infection.

## 2. Results and Discussion

### 2.1. ABBA Inhibits the Activity of S. aureus SrtA

To successfully establish an infection, bacteria produce a variety of virulence factors, such as surface proteins and secreted toxins [6,7,10]. For *S. aureus* and other Gram-positive bacteria, the anchorage of most surface proteins is indispensable, suggesting that the enzyme SrtA is an ideal target for the development of anti-virulence therapeutics [8,24]. Thus, the natural product, dryocrassin ABBA (ABBA, Figure 1A) has been screened as an effective agent against *S. aureus* SrtA activity using fluorescence resonance energy transfer (FRET). Consistent with previous results, the cleavage of the model substrate peptide Dabcyl-QALPETGEE-Edans (GL Biochem, Shanghai, China) through SrtA showed increasing fluorescence, detected using a microplate reader (Figure 1B). However, the addition of ABBA to the reaction system significantly decreased the fluorescence signal, indicating the inhibition of the catalytic activity of SrtA through ABBA (Figure 1B). Importantly, the IC_50_ of this inhibition was 24.17 μM (Figure 1B). Taken together, ABBA is an effective inhibitor of *S. aureus* SrtA, suggesting that this compound as a promising candidate against *S. aureus* infection.

### 2.2. Molecular Dynamics Simulation for SrtA-ABBA

**Figure 2:**
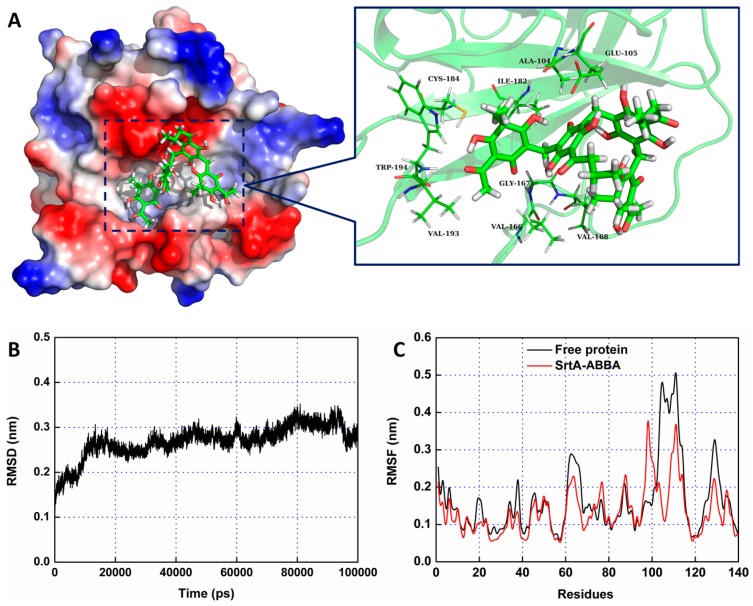
The 3D structural determination of the SrtA-ABBA complex using a molecular modeling method. (**A**) The structure of SrtA-ABBA; (**B**) The RMSD of the backbone atoms of the protein during MD simulations of SrtA-ABBA is presented; (**C**) The RMSF of the residue positions during the last 60-ns simulation with respect to the initial position of the SrtA protein in the free protein and complex systems.

To further characterize the molecular mechanism of ABBA against SrtA, the interaction between SrtA and ABBA was explored using molecular modeling. To indicate the convergence of the complex structure, the RMSD values of the SrtA in the whole system was examined as a function of time (Figure 2B). The backbone RMSD of SrtA reached equilibrium after ~20 ns of simulation, indicating that the 60-ns simulation applied in the present study was suitable for analysis. As shown in Figure 2A, the potential binding of ABBA to SrtA in the active site was determined using 100-ns molecular dynamics simulations. The results showed that ABBA binds to SrtA via intermolecular forces. In this complex system, ABBA localizes to the catalytic pocket of SrtA. Previous studies [25,26] have shown that the catalytic active region is the key position for SrtA, revealing that the side chain of ABBA forms a strong interaction with Glu105, Val166, Gly167, Val168, Trp194 and Val193, respectively. However, fluctuations of the residues in the binding sites of SrtA were explored based on root-mean-square fluctuations (RMSFs). As shown in Figure 2C, the fluctuation patterns of SrtA in the free protein and complex system are different during the final 50 ns of simulation. In this complex system, the residues (100–110, 120–140) in the binding site are bound, reflecting interactions with ABBA, thereby decreasing the degree of flexibility (the RMSF values less than 0.4 nm). However, the RMSF value in the free protein is precisely higher than that of complex system. Based on the above results, the residues Glu105/Val166/Gly167/Val168/Trp194/Val193 are the crucial binding sites for the stabilization of ABBA binding with SrtA. 

**Figure 3:**
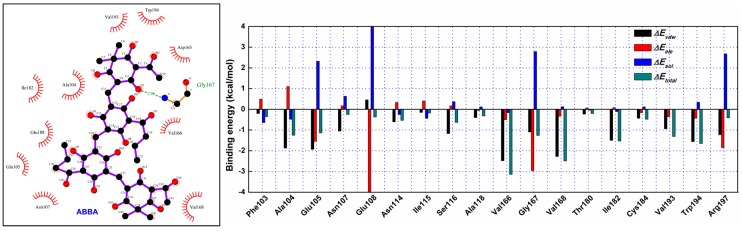
Decomposition of the binding energy on a per-residue basis at the binding sites of the SrtA-ABBA complex.

To identify the binding sites in the SrtA-ABBA complex, the MM-PBSA method was used to calculate the binding free energy between the residues surrounding the binding site and ABBA. As shown in Figure 3, an appreciable van der Waals (Δ*E_vdw_*) contribution of Val166 exists, with a Δ*E_vdw_* of ~−2.5 kcal/mol, reflecting the close proximity of the side chain of Val166 to the O3 atom of ABBA. Furthermore, Val168, Ile182 and Trp194 also have appreciable van der Waals interactions with ABBA because of the close proximity between the side chain residues and the O16, C41, O4, O3 and C8 atoms of ABBA. Interestingly, residue Gly167 showed an obvious electrostatic term (~−3.0 kcal/mol), while solvation (Δ*E_sol_*) showed unfavorable contributions at ~2.8 kcal/mol; consequently, the total energy contribution of Gly167 was strong (~−1.22 kcal/mol), indicating that a strong hydrogen bond exists between Gly167 and the O1 atom of ABBA. In addition, residues Ala104 and Glu105 also showed strong contributions to binding with ABBA, with Δ*E_vdw_* values of −1.89 and −1.91 kcal/mol (Figure 3). These results are consistent with the data shown in Figure 2, indicating that the residues Ala104/Glu105/Val166/Gly167/Val168/Ile182/Trp194 are key binding sites in the complex.

### 2.3. Validation of the Molecular Basis of ABBA against SrtA

**Figure 4:**
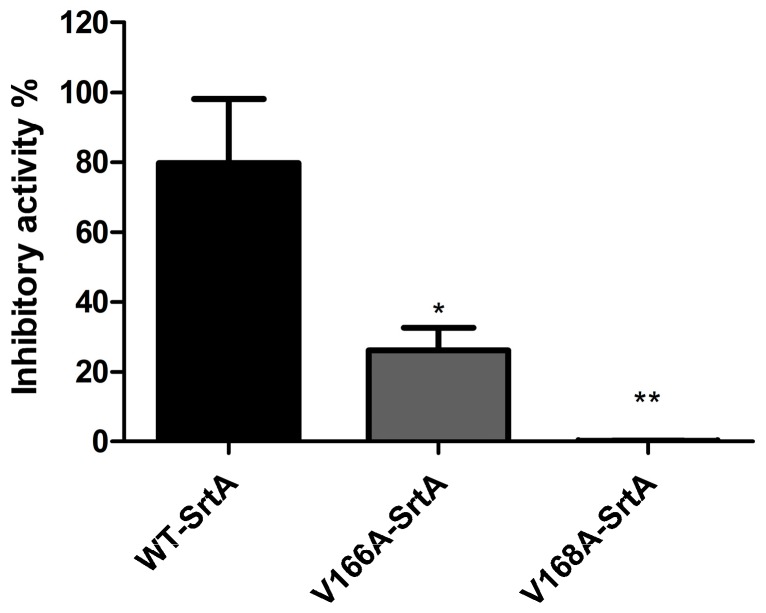
Inhibitory effects of ABBA against WT-SrtA and SrtA mutants. WT-SrtA and SrtA mutants (V166A-SrtA and V168A-SrtA) were incubated with 40 μM ABBA, and the catalytic activity of recombinant SrtA was determined as described in Figure 1B. The error bars show the standard deviations (SD). * *p* < 0.05, ** *p* < 0.01 compared with WT-SrtA.

Furthermore, to verify the accuracy of the binding site in the SrtA-ABBA complex, molecular modeling was performed for the two mutants, V166A-SrtA and V168A-SrtA, using the same procedure. The total binding free energy between WT-SrtA, SrtA mutants and ABBA was calculated to verify the accuracy of the above hypothesis using an MM-GBSA approach. As shown in Table 1, the binding energy of the mutants was weaker than that of WT-SrtA, with estimated Δ*G_bind_* values of −6.6 and −10.5 kcal/mol for V166A-SrtA and V168A-SrtA, respectively. Fluorescence spectroscopy quenching was used to measure the Δ*G_bind_* and the number of binding sites between ABBA and the two mutants, and these results were highly consistent with those obtained using computational methods (Table 1). For further validation of the simulation results, two mutants, V166A-SrtA and V168A-SrtA, were constructed, as shown in Figure 4, for FRET assay. As expected, the sensitivity of ABBA for these two mutants was significantly reduced compared with WT-SrtA (Figure 4). These results indicate that the information from the MD simulation on the SrtA-ABBA complex is reliable. The biological activity of SrtA was inhibited, reflecting the binding of the inhibitor, ABBA, to the active site region (residues of Ala104/Glu105/Val166/Gly167/Val168/Ile182/Trp194). 

## 3. Experimental Methods

### 3.1. Bacterial Strains, Growth and Reagents

ABBA was purchased from Shanghai Yuanye S&T Co., Ltd. (Shanghai, China). The solvent DMSO was obtained from Sigma-Aldrich (St. Louis, MO, USA). *S. aureus* strain USA 300 was used in the present study and cultivated at 37 °C in brain-heart infusion (BHI) broth (Sigma). GST affinity chromatography was used to purify the *S. aureus* recombinant SrtA from *E. coli* strain BL21 (DE3). The fluorescent peptide Dabcyl-QALPETGEE-Edans was purchased from GL Biochem (Shanghai, China).

### 3.2. Construction of Plasmids Encoding Wild-Type (WT)-SrtA, V166A-SrtA, V168A-SrtA

The DNA sequence encoding the WT-SrtA protein was amplified from *S. aureus* USA 300 genomic DNA and used as a template, with the primers (Table 2) to PCR-amplify the *S. aureus* SrtA sequence. Subsequently, the DNA fragment was digested with Nde1 and BamH1 and cloned into the pGEX-6P-1 expression vector. The sequence was verified through DNA sequencing, generating pGEX-6P-1-*srtA*, which encodes WT-SrtA. The site-directed mutagenesis of *S. aureus* SrtA (SrtA△N59), i.e., V166A-SrtA and V168A-SrtA, was realized using the QuickChange site-directed mutagenesis kit (Stratagene, La Jolla, CA, USA). The mutagenic primer pairs for the two mutations were listed in Table 2.

### 3.3. Expression and Purification of WT-SrtA, V166A-SrtA and V168A-SrtA

The recombinant plasmids, including WT-SrtA, were transformed into *Escherichia coli* BL21 (DE3) cells. Subsequently, the transformants were cultured and selected in Luria-Bertani (LB) media containing ampicillin (100 mg/L) at 37 °C, and 1 mM IPTG (Sigma) was added to induce protein expression when the OD600 reached approximately 0.6–0.8, followed by overnight culture at 16 °C. The bacteria were harvested through centrifugation at 5000 g for 20 min at 4 °C and resuspended in the reaction buffer (50 mM Tris-HCl, 5 mM CaCl_2_, and 150 mM NaCl, pH 7.5). The suspension was lysed through sonication to remove the cell debris, and the cell lysate was centrifuged at 10,000 g for 1 h at 4 °C. The supernatant was applied to a self-packed GST-affinity column (2 mL Glutathione-Sepharose 4B) (GE Amersham Biosciences, Piscataway, NJ, USA). After washing with the reaction buffer to remove the contaminating proteins, the GST-tagged protein was digested with Precision Protease at 4 °C overnight. Subsequently, the target protein was pooled using the reaction buffer. The point mutations V166A-SrtA and V168A-SrtA were expressed and purified using the same as applied for WT-SrtA.

### 3.4. Determination of Mutant and WT SrtA Activity

Fluorescence resonance energy transfer (FRET) was used to determine the activity of ABBA against WT-SrtA, V166A-SrtA and V168A-SrtA. As previously described [27], 100 μL of a mixture making up the reaction buffer, proteins and the corresponding agent gradients in 96-well plates was incubated for 30 min at 37 °C, followed by the addition of the fluorescent peptide substrate Dabcyl-QALPETGEE-Edans and incubation for 1 h at 37 °C to initiate the reaction. The fluorescence was read using emission and excitation wavelengths of 350 nm and 520 nm, respectively.

### 3.5. Molecular Modeling

In the present study, the 3D X-ray structure of SrtA (PDB code: 3CI5) was used for the molecular dynamics (MD) simulation. The software package AutoDock 4.0 (Molecular Graphics Laboratory, The Scripps Research Institute, La Jolla, CA, USA) was used to perform the standard docking procedure for a rigid protein and a flexible ligand. Following molecular docking, the molecular modeling of the SrtA with ABBA was performed, and the detailed processes for the computational biology method used in the present study have previously been described [28,29,30,31]. 

### 3.6. Binding Affinity Determination of ABBA with SrtA

In the present study, the binding constant (*K_A_*) of ABBA with SrtA was measured using a fluorescence-quenching method. A 280 nm excitation wavelength with a 5 nm band-pass and a 345 nm emission wavelength with a 10 nm band-pass were used to obtain the measurements. Details of the measurements have previously been described [32,33]. 

### 3.7. Statistical Analysis

The significance of the inhibitory activity was analyzed using SPSS 13.0 (SPSS Inc., Chicago, IL, USA) statistical software, with Student’s *t*-test, and the statistically significant differences indicated in the figures were considered when *p* < 0.05.

## 4. Conclusions

Here, we reported an effective SrtA inhibitor, ABBA, and determined the molecular mechanism of this inhibition. These results provide valuable information for the development of anti-virulence therapeutics for *S. aureus* infections.

## Figures and Tables

**Table 1:** The binding free energy (kcal/mol) of WT-LIG, V166A-LIG and V168A-LIG systems based on computational method and the values of the binding constants (*K_A_*) based on the fluorescence spectroscopy quenching.

Proteins	WT-SrtA	V166A	V168A
The binding energy	−25.8 ± 2.8	−19.2 ± 3.1	−15.3 ± 1.9
*K*_A_ (1 × 10^4^) L·mol^−1^	45.78 ± 6.5	43.5 ± 7.3	35.8 ± 5.5

**Table 2:** Oligonucleotide primers used in this study.

Primer Name	Oligonucleotide (5–3) ^a^
WT-SrtA-F	GCGGGATCCCAAGCTAAACCTCAAATTCC
WT-SrtA-R	CCGCTCGAGTTATTTGACTTCTGTAGCTACAA
V166A-SrtA-F	GTTAAGCCTACAGATGCGGGAGTTCTAGATGAAC
V166A-SrtA-R	GTTCATCTAGAACTCCCGCATCTGTAGGCTTAAC
V168A-SrtA-F	CCTACAGATGTAGGAGCGCTAGATGAACAAAAAGG
V168A-SrtA-R	CCTTTTTGTTCATCTAGCGCTCCTACATCTGTAGG

^a^ Restriction endonuclease recognition sites or mutated codons are underlined.
